# The Effect of Different Particle Sizes of Fly Ash on the Properties of Mortar

**DOI:** 10.3390/ma18204693

**Published:** 2025-10-13

**Authors:** Changqing Wu, Yuanquan Yang, Bo Pang, Yunpeng Cui

**Affiliations:** School of Civil Engineering, Liuzhou Institute of Technology, Liuzhou 545616, China; wuchangqing@lzhit.edu.cn (C.W.); yangyuanquan@sylu.edu.cn (Y.Y.); pangbo0829@sylu.edu.cn (B.P.)

**Keywords:** fly ash, particle size distribution, cement mortar, compressive strength, pore structure distribution

## Abstract

Fly ash is a commonly used mineral addition in construction engineering. Research on its different particle size distributions can help optimize material performance, promote resource utilization, and support environmental protection. In this study, the particle size of fly ash was used as a variable; fly ash with a single particle size was prepared by means of sieving, and the particle size was precisely controlled as a variable, thus avoiding the errors caused by the addition of multiple different particle sizes. Replacing 10% of the cement with fly ash to prepare cement mortar, the influence of fly ash particle size on the performance of cement mortar was investigated. The results show that the mortar incorporating fly ash with a particle size range of 10–20 μm achieves a 28-day compressive strength of 58.25 MPa and a flexural strength of 10.29 MPa. The hydration heat release rate of fly ash in the 10–20 μm range reaches a maximum of 1.84 mW/g, and the total hydration heat release peaks at 211.17 J/g at 70 h. The influence of fly ash particle size on the total hydration heat release is relatively small in the early stages but increases rapidly with prolonged hydration time. When the fly ash particle size is in the 10–20 μm range, the cement mortar exhibits the lowest total porosity at 12.88%, with the smallest average pore size of 27.1 nm and the smallest most probable pore size of 21.2 nm. This reduces harmful pores, increases the number of harmless pores, makes the cement mortar structure denser, and improves the durability of the mortar. The types of hydration products of different particle sizes of fly ash did not change. The smaller the particle size of fly ash, the more complete the volcanic ash reaction, promoting the hydration of mortar.

## 1. Introduction

Fly ash, a predominant solid waste generated from coal-fired power plants, is commonly utilized as a supplementary cementitious material (SCM) in mortar formulations. It partially replaces Portland cement and is also incorporated into blended Portland cement production [1,2,3]. In Chinese thermal power plants, the primary oxide constituents of fly ash include SiO_2_, Al_2_O_3_, FeO, Fe_2_O_3_, CaO, TiO_2_, MgO, K_2_O, Na_2_O, SO_3_, and MnO_2_ [4]. The abundant Na^+^ and K^+^ ions in fly ash react with hydration products like Ca(OH)_2_ to form calcium silicate (Ca-Si) and calcium aluminate (Ca-Al) compounds, effectively enhancing mortar strength through pore-filling mechanisms [5,6,7,8]. As a mortar admixture, it significantly improves the workability and durability of mortar while mitigating the heat of hydration generated by cement hydration through the following mechanisms: physical filling and particle gradation effects, morphological effects and ball-bearing lubrication, hydration kinetics regulation, and rheological property enhancement mechanisms [9]. Moreover, its utilization in building materials substantially reduces land occupation for waste storage, achieving dual benefits of waste valorization and environmental conservation through resource efficiency [10,11,12].

To optimize mortar performance, enhancing the pozzolanic activity of fly ash remains crucial. Conventional mechanical grinding techniques, while effective, present two critical limitations: increased energy consumption during preprocessing and uncontrollable particle size distribution of processed fly ash. Fineness emerges as the most critical parameter governing fly ash performance in Portland cement-based systems [13,14,15,16]. Typical fly ash particles range up to 200 μm, with approximately 75% of particles below 45 μm. The material density varies between 2.2 and 2.7 g/cm^3^, while specific surface area approximates that of cement, contingent on separation processes [17,18,19,20]. Previous studies demonstrate strong correlations between fly ash fineness or particle size distribution and key mortar properties including strength, wear resistance, and freeze–thaw durability [21,22,23,24,25]. Notably, coarser particles exhibit decreased SiO_2_, Fe_2_O_3_, MgO, SO_3_, and K_2_O content but increased Al_2_O_3_, CaO, and Na_2_O concentrations [26,27,28]. Excessive fineness reduction may elevate hydration heat release, impair workability, and increase water absorption [29]. Optimizing particle size distribution therefore presents significant potential for expanding fly ash applications in cementitious systems while enhancing environmental benefits [30,31,32].

Current research predominantly focuses on mechanical grinding approaches to investigate the effects of fly ash fineness and particle size distribution on mortar properties. However, such methods inherently produce poorly controlled particle size distributions, limiting precise design optimization of mortar performance [33]. This study innovatively employs fly ash with distinct particle size ranges (60–80 μm, 30–50 μm, and 10–20 μm), fly ash with a single particle size is prepared by means of sieving, and the particle size is precisely controlled as a variable, thus avoiding the errors caused by the addition of multiple different particle sizes. It was found through preliminary tests that the mortar exhibited the best performance when the fly ash dosage was 10%. To eliminate the cross-interference between the two variables of “dosage” and “particle size”, an optimal dosage was fixed as the research benchmark. Therefore, this study was conducted under the condition that fly ash was used as a substitute for 10% Portland cement. Systematic investigations are conducted to elucidate the impacts of both absolute particle size and size distribution parameters on mortar mechanical properties, hydration thermal characteristics, phase evolution, pore structure, and microstructural development.

## 2. Materials and Testing Methods

### 2.1. Materials

**Figure 1:**
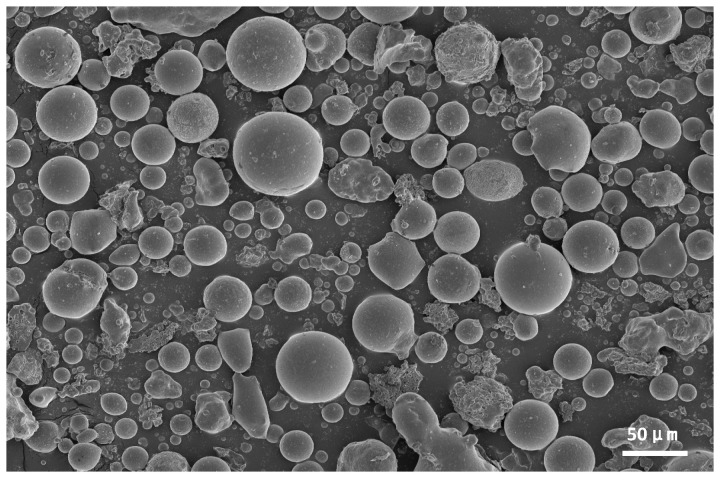
Microstructure of fly ash.

In this study, P·O 42.5 ordinary Portland cement (OPC) produced by Shenyang Jidong Cement Co., Ltd. (Shenyang, China) was used. The composition analysis of the cement is shown in Table 1, and the basic properties of the cement are shown in Table 2. The fine aggregate was natural river sand with a fineness modulus of 2.6, the average particle size is 0.5 mm. Grade I fly ash was selected, which was produced by CGN Innovation New Materials Co., Ltd. (Yichang, Hubei, China) and had a uniform particle size distribution. The composition analysis and physical properties indicators of fly ash are shown in Table 3 and Table 4, and the microstructure of fly ash is shown in Figure 1.

### 2.2. Mix Proportion

In this study, 10% Portland cement was replaced with fly ash in different particle size ranges after screening. The water cement ratio is 0.4. Mortar mix is shown in Table 5.

### 2.3. Testing Methods

#### 2.3.1. Preparation of Fly Ash with Different Particle Size

**Figure 2:**
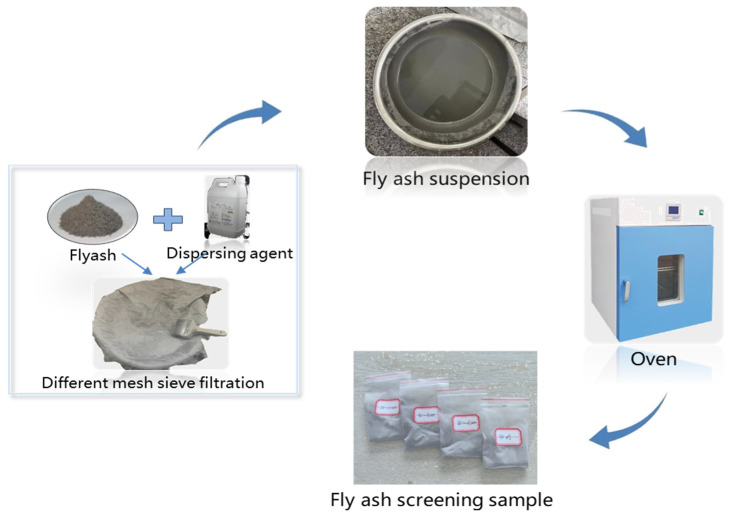
Preparation process of fly ash with different particle size.

Fly ash was fractionated into distinct particle size ranges using standardized sieves, with the corresponding mesh sizes and particle diameters detailed in Table 6. The fractionation procedure commenced by mounting a 180-mesh sieve over a container, followed by pouring raw fly ash onto the sieve and mechanically separating particles using a brush. Particles passing through the 180-mesh sieve but retained on the 250-mesh sieve constituted the 80–60 μm fraction. For particles < 60 μm requiring enhanced sieving efficiency due to insufficient gravitational settling, ethanol (≥95% purity) was employed as a dispersion agent to mitigate particle agglomeration and expedite sieving. This protocol yielded the 50–30 μm fraction (passing 300-mesh but retained on 500-mesh sieves) and the 20–10 μm fraction (passing 800-mesh but retained on 1400-mesh sieves) as aqueous suspensions. These suspensions underwent low-temperature drying at 60 °C in an oven. Ethanol was specifically selected as an inert dispersion medium that neither chemically interacts with fly ash constituents nor alters their inherent properties, while effectively preventing particle aggregation in solution. The entire particle size fractionation workflow is schematically illustrated in Figure 2.

#### 2.3.2. Preparation of Mortar

Following standard JGJ/T70-2009 “Standard for Test Methods of Basic Properties of Construction Mortar” [34], mortar specimens were prepared using the prescribed mix proportions. The mixed mortar was poured into 40 mm × 40 mm × 160 mm molds after mechanical blending in a mortar mixer. Vibratory compaction was achieved through a standard mortar vibrating table. Before demolding, the test sample is cured for 24 h under controlled laboratory conditions (20 ± 2 °C, RH ≥ 95%). After demolding, they were subjected to extended curing under the same conditions until reaching the specified ages of 3 days, 7 days, and 28 days. Mechanical property testing and material characterizations were subsequently conducted at each curing interval. This protocol ensured standardized evaluation of compressive strength, flexural strength, and other performance metrics in accordance with established cementitious material testing methodologies.

### 2.4. Characterization Method

#### 2.4.1. Mechanical Properties

The TYE-300 compression testing machine was used to test the compressive and flexural strength of fly ash cement mortar. The tests involved taking the average of three specimens of each fly ash cement mortar, tested at curing periods of 3, 7, and 28 days.

#### 2.4.2. Setting Time Test

The setting time of FA mortar is measured using a Vicat apparatus, testing both the initial and final setting times. It follows GB/T 1346 [35] Methods for Testing the Water Requirement of Standard Consistency, Setting Time, and Stability of Cement.

#### 2.4.3. Granularity Distribution

Malvern laser particle size analyzer (Bettersize 2000 is produced by Liaoning Dandong Better Instrument Co., Ltd., Dandong, China) was used to determine the particle size distribution of the material in each particle size range. The specific parameters are: the refractive index of the solid particle is 1.58, the refractive index of the dispersion medium is 1.29, and the shading is about 13%.

#### 2.4.4. Rheological Property

The rheological properties were measured using an LBY-III mortar rheometer with an SC4-21 rotor, produced by Boying Technology Co., Ltd. in Beijing, China. Each set of samples was tested for 10 min. The shear rate range was set from 0 to 30 s^−1^ to determine the plastic viscosity, shear stress, and yield stress of the Fly Ash Cement Mortar.

#### 2.4.5. XRD

In the experiment, Ultima IV X-ray diffractometer and Cu-Kα radiation (1.541874 Å, 40 kV, 40 mA) were used for phase analysis. When testing fly ash with different particle sizes, fly ash was placed in a 45 °C vacuum drying oven for 48 h, and then scanned at a scanning speed of 8°/min, a step of 0.02°, and a continuous scanning method. When the hydration products were tested, the samples were taken after curing to the specified age, immediately soaked in anhydrous ethanol for 24 h, and then dried in a 45 °C vacuum drying oven for 48 h, and then the samples were placed in a grinding bowl. Grinding and passing through a 200 mesh sieve, and then scanning at a speed of 8°/min, a step of 0.02°, continuous scanning mode for scanning.

#### 2.4.6. FT-IR

The infrared spectrometer used is Thermo Fisher Nicolet iS50, produced by Thermo Fisher Scientific Co., Ltd. in Shanghai, China. Firstly, fly ash with different particle sizes was mixed with potassium bromide powder after drying and grinding, and the mixing ratio of potassium bromide to the sample was 100:1. The mixed samples were pressed into thin sheets with a diameter of 12 mm and a thickness of about 0.4 mm by a tablet press. The spectral scanning range is 4000 cm^−1^–400 cm^−1^.

#### 2.4.7. SEM

The QUANTA 450 SEM (FEI Co., Hillsboro, OR, USA) was used for testing. The fly ash with different particle sizes was put into a vacuum drying oven at 45 °C for 48 h, and the sample was placed on a conductive tape. Then the samples were sprayed with gold, and finally the SEM microstructure analysis was performed at different magnifications, and the EDS test was performed at the specified position. The specific parameters are as follows: voltage is 230 V, frequency is 50/60 Hz, current is 8 A; the accelerating voltage range is continuously adjustable between 200 V and 30 kV.

#### 2.4.8. Hydration Heat

The test was carried out using a TAM-air instrument. In detail, 40 mg of cement and fly ash were weighed and mixed evenly into a 20 mL ampoule, and the water into the syringe. The ampoule were then placed in a TAM-air instrument at a temperature of 20 °C. The water from the syringe was injected into the ampoule and stirred for 2 min with the inbuilt stirrer after the instrument was stabilized, while data collection was started.

#### 2.4.9. MIP

In this experiment, AUTOPORE IV 9500 mercury porosimeter produced by Huapeng General Technology Co., Ltd. in Shenzhen, China was used to test the pore size characteristics of the samples. Before the test, the samples reaching the age were dried at 70 °C for 5 h, and about 1.5 g–2.5 g of the samples were taken and analyzed under low pressure and high pressure, respectively.

#### 2.4.10. TG/DSC

In this experiment, the TG/DSC analyzer (Netzsch STA 449 F3, Seeburg, Germany) was used for detection. A paste sample with a mass of approximately 10 mg was weighed and placed in an alumina crucible. The temperature was raised from 20 °C to 1000 °C at a heating rate of 10 °C/min.

## 3. Results and Discussion

### 3.1. Characteristics of Fly Ash with Different Particle Sizes

#### 3.1.1. Particle Size Distribution of Fly Ash

**Figure 3:**
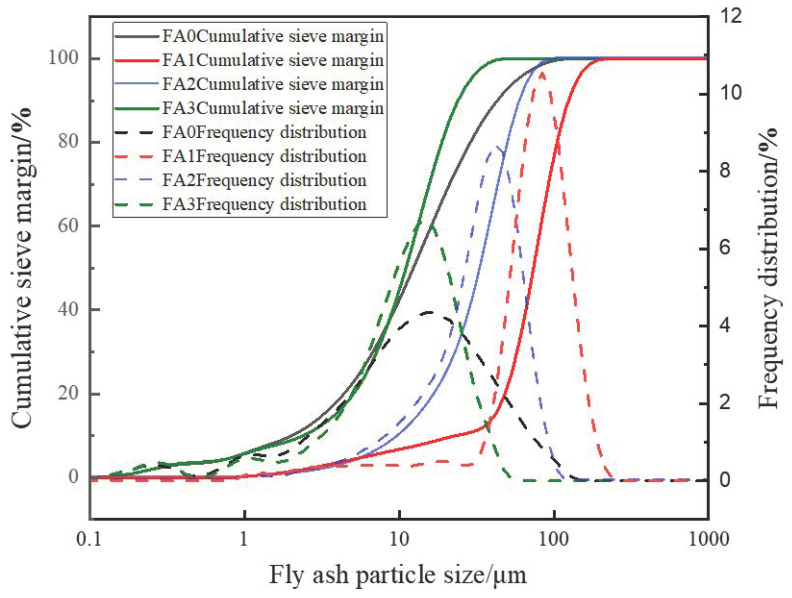
Distribution of different particle sizes of fly ash after screening.

The particle size distribution of fly ash after sieve screening is shown in Figure 3. From Figure 3, it can be seen that the median diameter D50 of FA0 fly ash is about 30 μm, the median diameter D50 of FA1 fly ash is about 70 μm, the median diameter D50 of FA2 fly ash is about 40 μm, and the median diameter D50 of FA3 fly ash is about 15 μm. The particle size distribution test results verify that the average particle size of the above fly ash meets the requirements of this experimental standard.

#### 3.1.2. XRD Analysis of Fly Ash with Different Particle Size

**Figure 4:**
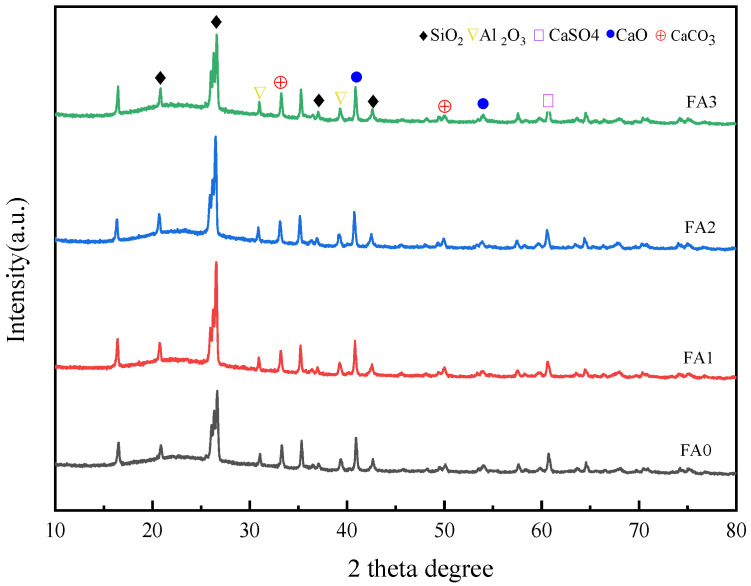
XRD analysis diagram of fly ash with different particle sizes.

The XRD patterns (Figure 4) demonstrate compositional homogeneity across different particle size fractions, indicating that the mineralogical composition of fly ash remains relatively stable regardless of particle dimensions. The predominant mineral phases identified include SiO_2_, Al_2_O_3_, CaO, CaCO_3_, and CaSO_4_, which are characteristic constituents of fly ash. Notably, SiO_2_ and CaO exhibit pronounced diffraction peak intensities, reflecting their higher crystallinity. The well-crystallized SiO_2_, a dominant mineral phase in fly ash, likely originates from the combustion process of coal, where thermal conditions favor the formation of crystalline silica. Similarly, the distinct crystallization of CaO may be attributed to high-temperature exposure during combustion, which facilitates oxide crystallization. In contrast, Al_2_O_3_ displays relatively weaker diffraction peaks, suggesting lower crystallinity. This phenomenon could stem from the complex speciation of aluminum in fly ash, potentially existing in multiple forms such as aluminosilicates or alumina oxides, with a significant proportion present in amorphous phases, thereby reducing overall crystallinity.

During fly ash formation, substantial emissions of SO_2_ and CO_2_ gases occur during coal combustion. These reactive gases interact with CaO within the fly ash through gas–solid reactions, leading to the formation of CaCO_3_ and CaSO_4_. Collectively, the XRD analysis provides critical insights into the mineralogical composition and crystallinity characteristics of fly ash, offering a scientific foundation for its effective utilization. In practical applications, the selection of optimal utilization strategies-whether as SCM, geopolymer precursors, or construction aggregates-should be guided by a comprehensive understanding of fly ash’s mineralogical profile and physicochemical properties. Such knowledge-driven approaches enable the maximization of its technical performance and environmental benefits, promoting sustainable resource utilization in construction and materials engineering.

#### 3.1.3. FT-IR Analysis of Fly Ash with Different Particle Size

**Figure 5:**
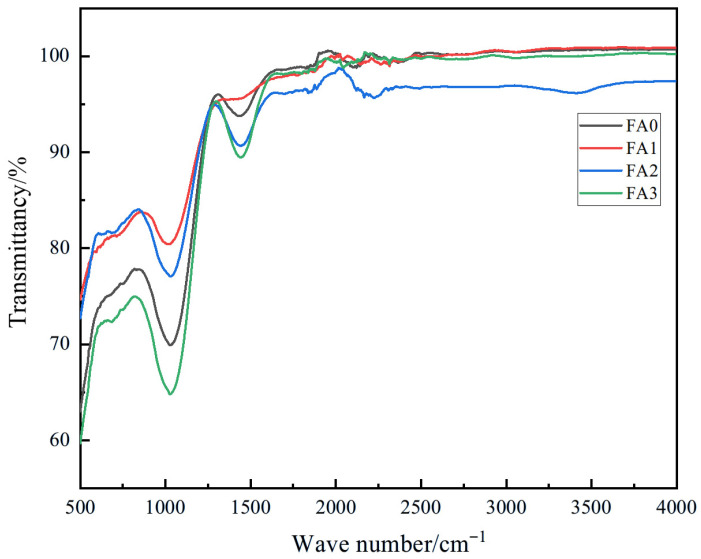
FT-IR analysis diagram of fly ash with different particle sizes.

FT-IR analysis of fly ash with different particle sizes is presented in Figure 5. This technique identifies characteristic absorption peaks in the infrared spectrum to determine the chemical composition of fly ash. The FT-IR spectra reveal two prominent absorption bands near 1100 cm^−1^ and 1450 cm^−1^ across all particle size fractions. These bands correspond to specific bond vibrations of the primary constituents in fly ash. The 1100 cm^−1^ band is attributed to the Si-O stretching vibrations in SiO_2_, confirming the abundance of silica in fly ash. The 1450 cm^−1^ band corresponds to Al-O stretching vibrations in Al_2_O_3_, indicating the presence of aluminum oxide in significant quantities.

Notably, the FA3 exhibited the lowest transmittance in the FT-IR spectra. Reduced transmittance reflects stronger light absorption or scattering by the material, which correlates with enhanced bond energy of Si-O bonds in SiO_2_. Bond energy, a measure of chemical bond stability, increases with stronger bonds, rendering them more resistant to disruption. The dominance of Si-O bonds in SiO_2_ suggests improved structural stability of silica in finer fly ash particles.

Furthermore, the transmittance decreases progressively with reduced particle size, a phenomenon also linked to increased bond energy of Al-O bonds in Al_2_O_3_. This inverse correlation demonstrates that smaller particle sizes enhance the stability of Al_2_O_3_ within fly ash. The combined FT-IR and particle size analysis underscores the critical role of particle dimension in modulating the chemical stability and optical behavior of fly ash constituents, providing insights for optimizing its performance in material applications.

#### 3.1.4. SEM Analysis of Fly Ash with Different Particle Size

**Figure 6:**
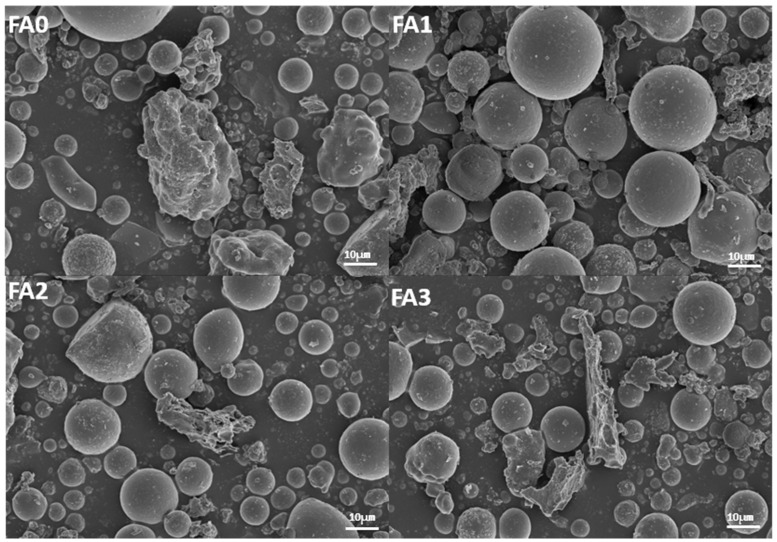
SEM scanning images of fly ash with different particle sizes.

SEM analysis was conducted at a magnification of 3000× to evaluate particle size, morphology, and distribution across different fly ash fractions, as illustrated in Figure 6. The SEM micrographs reveal diverse surface morphologies of fly ash particles, including spherical, ellipsoidal, and irregular geometries, reflecting morphological variations induced by sieving processes. Particles in the unsieved control group (raw fly ash) predominantly exhibited irregular shapes, with dimensions reaching approximately 100 μm in length and 50 μm in width, indicating inherent morphological complexity and heterogeneity.

In contrast, sieved fly ash fractions demonstrated significant morphological refinement. Both coarse and fine sieved fractions displayed uniformly distributed spherical particles with smooth surfaces, diverging markedly from the irregular, rough-surfaced particles observed in the control group. This morphological divergence underscores the substantial impact of particle size classification on fly ash morphology. Post-sieving, particle dispersion improved significantly due to effective segregation of distinct size fractions, which minimized interparticle agglomeration and overlapping. The resultant homogeneous particle distribution highlights the efficacy of sieving in optimizing morphological uniformity, a critical factor influencing material performance in cementitious and geopolymer applications.

### 3.2. The Effect of Fly Ash Particle Size on the Properties of Cement Mortar

#### 3.2.1. Effect of Different Particle Size Fly Ash on the Mechanical Properties of Mortar

**Figure 7:**
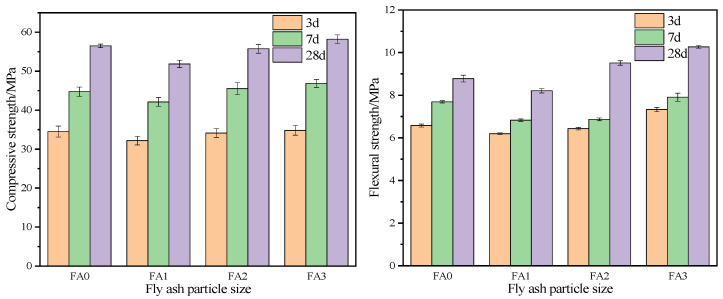
Relationship between particle size of fly ash and mechanical properties of mortar.

Fly ash particles with small particle size can more effectively fill the gap between cement particles, reduce the porosity of cement mortar and improve the compactness of mortar. This filling effect helps to improve the compressive strength and flexural strength of cement mortar. The compressive and flexural strength tests were measured by 40 mm × 40 mm × 160 mm test blocks, and the compressive and flexural strength values of the test blocks were measured for 3 days, 7 days and 28 days, respectively. The compressive and flexural strength values of cement mortar at different ages of FA0, FA1, FA2 and FA3 are shown in Figure 7.

Analysis of Figure 7 reveals distinct variations in mechanical strength between mortars incorporating sieved fly ash fractions (with different particle size ranges) and unsieved fly ash at curing ages of 3, 7, and 28 days. The compressive strength results demonstrate an initial increase with decreasing particle size during early curing stages. At 3 days, FA3 specimens (20–10 μm) exhibited a 1.8% higher compressive strength compared to FA0, with further increases of 3.3% and 4.6% observed at 7 and 28 days, respectively. However, the compressive strength differences among the three particle size fractions became negligible at the 28-day curing age.

In contrast, flexural strength showed sustained enhancements across all curing stages for the finest fraction (20–10 μm). FA3 specimens achieved the highest flexural strength values, surpassing FA0 by 11.8% at 3 days, 2.8% at 7 days, and 16.4% at 28 days. This performance enhancement arises from the synergistic effects of particle packing and pozzolanic activity. The optimized particle size distribution (20–10 μm) promotes efficient mechanical packing while facilitating the formation of calcium silicate hydrate (C-S-H) gel through pozzolanic reactions, collectively improving matrix densification.

The superior flexural strength of FA3 specimens across all curing ages directly correlates with the refined particle packing effect, which minimizes void spaces in the mortar matrix. When fly ash particles function similarly to fine aggregates, their optimized size distribution enables effective filling of interstitial spaces between cement grains, thereby achieving high compactness. These findings underscore the critical role of particle size distribution in governing filler efficiency.

The results demonstrate that strategic particle size optimization allows for higher Portland cement replacement rates with fly ash, thereby advancing waste valorization in construction materials. This approach not only enhances environmental sustainability by reducing cement consumption but also improves cost-effectiveness through optimized material utilization. The study provides a technical foundation for tailoring fly ash particle characteristics to maximize performance benefits in cementitious composites.

#### 3.2.2. The Effect of Fly Ash Particle Size on the Setting Time of Cement Mortar

**Figure 8:**
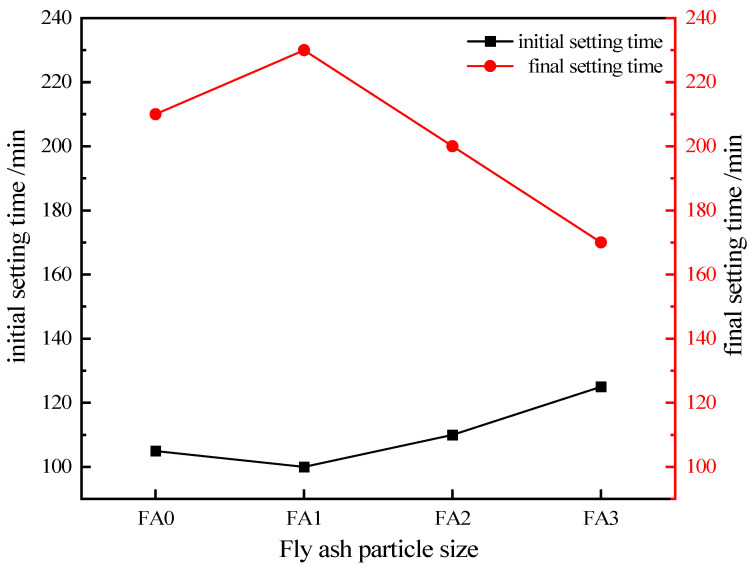
Trend of variation in setting time of fly ash with different particle sizes.

Figure 8 presents the setting time of cement mortar under the condition of fly ash with different particle sizes. As the particle size of fly ash decreases, the initial setting time of the mortar is prolonged, while the final setting time is shortened. Among them, compared with FA0, the initial setting time of FA3 increased by 19.0%, while its final setting time decreased by 19.1%. The smaller the particle size of fly ash, the larger its specific surface area. This not only enables the pozzolanic effect to promote the hydration reaction of cement mortar, but also allows the smaller fly ash particles to exert a filling effect in the mortar—both of which contribute to shortening the final setting time of cement mortar.

#### 3.2.3. Effect of Fly Ash with Different Particle Sizes on the Rheological Properties of Cement

**Figure 9:**
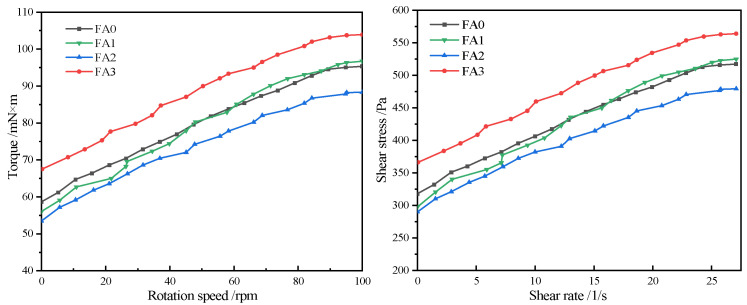
Relationship between Shear Rate and Shear Stress in Fly Ash Mortar with Different Particle Sizes.

Table 7 presents the rheological properties of mortar incorporating fly ash with different particle sizes. As the particle size of fly ash decreases, both the yield stress and plastic viscosity of the fly ash mortar exhibit a trend of first increasing and then decreasing. Figure 9 shows the relationships between rotational speed-torque and shear rate-shear stress of mortar with fly ash of different particle sizes. As the rotational speed and shear rate increase, both the torque and shear stress increase accordingly. When the shear stress reaches the maximum value, the cement mortar specimen will undergo plastic deformation. All samples exhibit a linear relationship that does not pass through the origin in terms of rotational speed-torque and shear rate-shear stress changes, belonging to the Bingham fluid rheological type. This indicates that the incorporation of fly ash with different particle sizes does not alter the rheological type of the mortar, but only changes the indices of yield stress and plastic viscosity. Most of the C-S-H formed during the cement hydration process undergoes flocculation. The incorporation of a certain amount of fly ash can improve the fluidity of the slurry. When the particle size of fly ash decreases, it can better fill the pores in the mortar, improve the compactness and uniformity of the mortar, which in turn leads to an increase in the water demand of the mortar and a decrease in yield stress and plastic viscosity. As the particle size of fly ash decreases, both the yield stress and plastic viscosity of the cement mortar gradually decrease.

#### 3.2.4. Effect of Fly Ash with Different Particle Size on Hydration Heat of Cement

**Figure 10:**
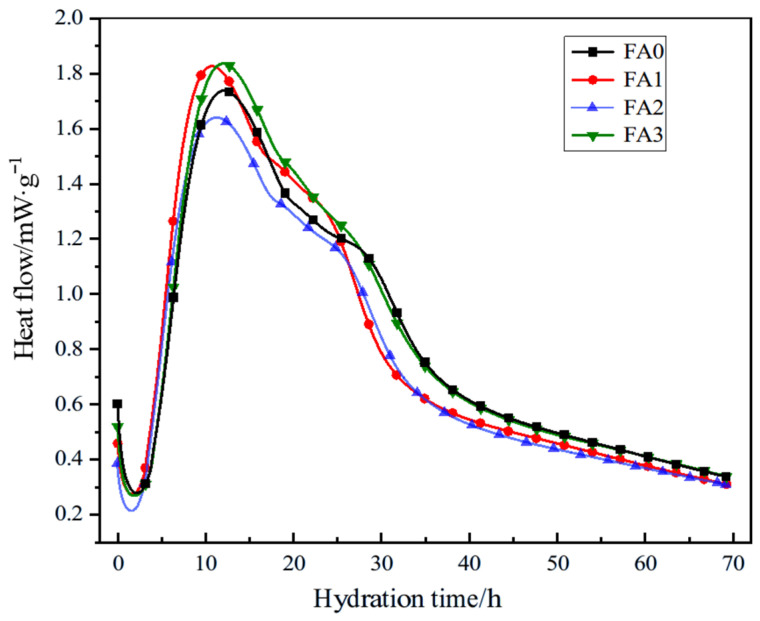
Effect of different particle sizes of fly ash on the heat release rate of cement hydration.

**Figure 11:**
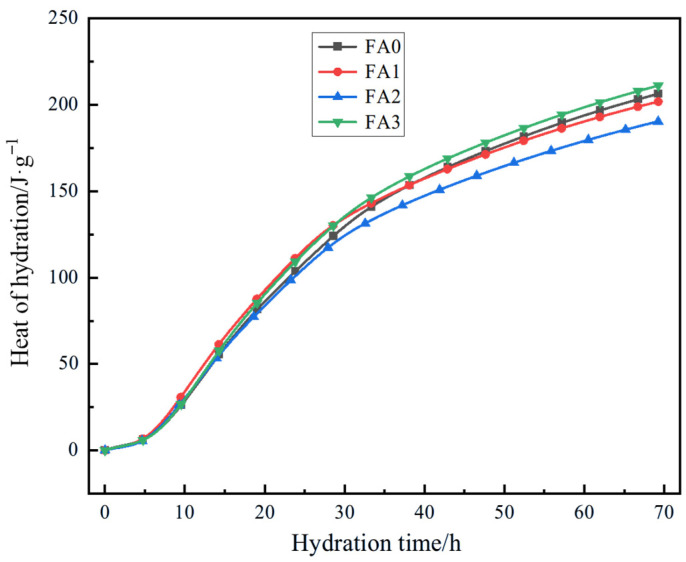
Effect of different particle size of fly ash on hydration heat of cement.

Fly ash delays the initial hydration of cement in cement mortar, and reduces the free water loss caused by cement hydration in cement mortar. On the other hand, fly ash has a large specific surface area and can adsorb a part of free water. With the hydration of cement, the adsorbed water is gradually released, which supplements the water content in cement mortar. In the later stage, fly ash will react with Ca(OH)_2_ generated in the hydration process of cement to form secondary hydration products such as C-S-H gel. In order to further study the effect of particle size distribution of fly ash on the hydration heat of cement mortar, fly ash with different particle size distribution ranges was selected to replace 10% cement to study the hydration heat release process. The hydration heat release rate is shown in Figure 10, and the hydration heat release is shown in Figure 11.

Analysis of Figure 10 reveals significant differences in hydration heat release rates among cement pastes incorporating fly ash of distinct particle sizes. During the 0–4 h induction period, the minimum hydration heat release rates were recorded as follows: 0.22 mW/g for FA2, 0.28 mW/g for FA0, 0.27 mW/g for FA3, and 0.27 mW/g for FA1. Notably, FA2 exhibited a 21.43% reduction in heat release rate compared to the control group.

In the accelerated hydration phase, peak heat release rates occurred at approximately 12 h, with FA3 demonstrating the highest value of 1.84 mW/g, followed by FA1 (1.83 mW/g), FA0 (1.74 mW/g), and FA2 (1.64 mW/g). The FA3 fraction showed a 5.75% increase in peak heat release rate compared to FA0. The maximum observed difference in hydration heat release rates reached 0.20 mW/g, corresponding to 11.50% of the control group’s peak value, demonstrating the significant influence of fly ash particle size on cement hydration kinetics.

These variations likely stem from particle size-dependent effects on nucleation sites for hydration products and pozzolanic reactivity. Finer particles provide increased surface area for heterogeneous nucleation of calcium silicate hydrate (C-S-H) phases, accelerating early hydration, while coarser fractions may delay reaction kinetics due to reduced interfacial activity. The results highlight the critical role of particle size optimization in tailoring cement hydration behavior for specific thermal management requirements in mass concrete applications.

Analysis of Figure 11 demonstrates distinct variations in cumulative hydration heat generation influenced by fly ash particle size. During the initial 10 h of hydration, all fly ash fractions exhibited minimal impact on cement hydration, with low cumulative heat release. However, cumulative heat generation accelerated significantly with prolonged hydration time, revealing pronounced particle size-dependent effects. At 70 h, the FA3 fraction achieved the highest cumulative heat of 211.17 J/g, followed by FA0 at 206.40 J/g, FA1 at 201.91 J/g, and FA2 with the lowest value of 190.43 J/g. Post-70 h, hydration heat accumulation plateaued, indicating negligible further hydration activity, which was not investigated in this study.

Compared to the control group, FA2 reduced cumulative heat generation by 7.74%, while FA1 showed a smaller reduction of 2.18%. Conversely, FA3 increased cumulative heat by 2.31%, suggesting enhanced hydration activity. These observations highlight the critical role of fly ash particle size in modulating cement hydration thermodynamics. Finer fly ash particles possess a larger specific surface area, which increases interfacial contact with water, accelerates reaction kinetics, and promotes higher heat release. Conversely, coarser particles reduce surface reactivity, thereby suppressing heat generation.

As a heterogeneous material, fly ash exhibits significant differences in composition and physical properties after classification. FA2 is exactly at the critical particle size threshold, which results in a lower total hydration heat release compared to FA0. In terms of composition, FA2 may have accidentally accumulated a higher proportion of hydration-inert phases or unburned carbon, leading to a reduction in the total hydration heat release. From the perspective of physical effects, compared with FA0, FA2 has a larger specific surface area, which enhances hydration activity; however, its particle morphology tends to form unfavorable packing structures. These structures inhibit water penetration and ion diffusion, hinder the hydration process, and consequently result in a lower total hydration heat release.

### 3.3. Effect of Different Particle Size Fly Ash on Pore Structure of Mortar

#### 3.3.1. Effect of Fly Ash Particle Size on the Porosity of Mortar

**Figure 12:**
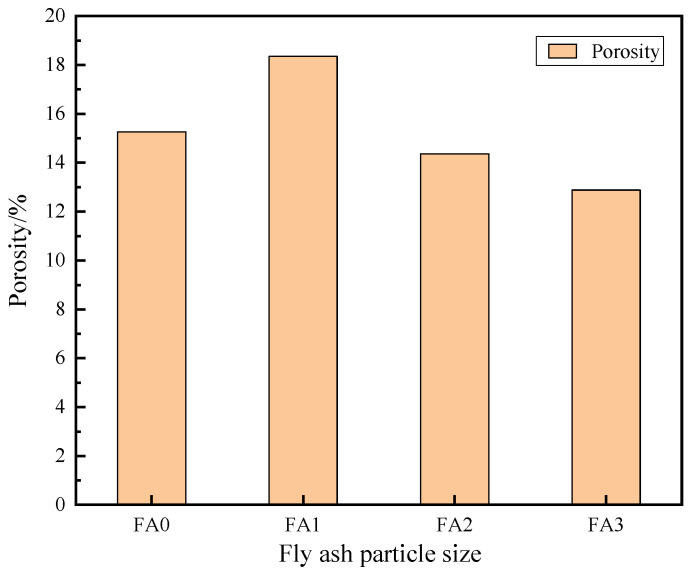
Effect of different particle sizes of fly ash on the porosity of cement mortar.

The influence of fly ash with different particle sizes of FA0, FA1, FA2 and FA3 on the porosity of cement mortar at a maintenance age of 28 days is shown in Figure 12. it can be seen that the total porosity of fly ash mortar of FA0 is 15.26%, the total porosity of fly ash mortar of FA1 is 18.35%, which is 20.3% higher than FA0, the total porosity of fly ash mortar of FA2 is 14.36%, which is 5.9% lower than FA0, and the total porosity of fly ash mortar of FA3 is 12.88%, which is 15.6% lower than FA0. In general, the total porosity of mortar decreases with the decrease in fly ash particle size. The particle size of fly ash has a great influence on the total porosity of mortar. With the decrease in fly ash particle size, the specific surface area increases, and the chemical activity also increases accordingly. It is helpful to generate more hydration products, such as hydrated calcium silicate and other gel substances in the process of cement hydration. These gel materials can fill the original pores, thereby reducing the porosity of the hardened paste.

#### 3.3.2. Effect of Fly Ash Particle Size on the Average Pore Size and the Most Probable Pore Size of Mortar

**Figure 13:**
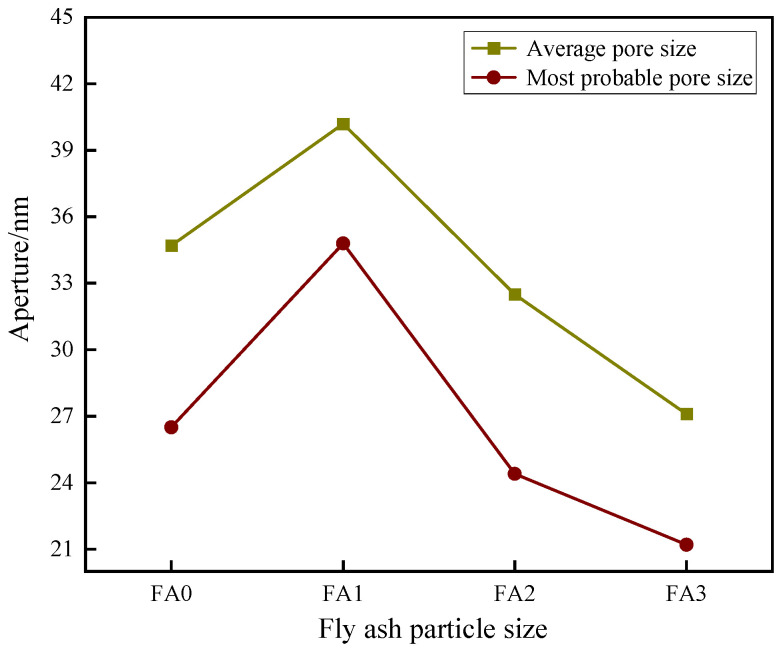
Effect of different particle sizes of fly ash on the pore size of cement mortar.

Figure 13 presents the effect of fly ash with different particle sizes (FA0, FA1, FA2, and FA3) on the pore structure of cement mortar at a maintenance age of 28 days. The results demonstrate that the mortar containing unsieved fly ash exhibits an average pore diameter of 34.7 nm and a most probable pore diameter of 26.5 nm. In contrast, FA1 modified mortar shows increased pore sizes, with the average and most probable pore diameters reaching 40.2 nm and 34.8 nm, representing 15.9% and 31.3% increases compared to FA0, respectively. Conversely, mortars incorporating finer fly ash fractions display reduced pore dimensions: FA2 yields average and most probable pore diameters of 32.5 nm (6.3% decrease) and 24.4 nm (7.9% decrease), while FA3 demonstrates the most significant refinement with values of 27.1 nm (21.9% decrease) and 21.2 nm (20.0% decrease), respectively.

The experimental data reveal a consistent trend where both average and most probable pore diameters decrease with diminishing fly ash particle size. This phenomenon can be attributed to the effective pore-filling capability of fine fly ash particles, particularly those in the submicron range, which occupy the microscopic voids within the cementitious matrix. The incorporation of appropriately sized fly ash particles optimizes the pore size distribution, leading to enhanced matrix densification. Notably, the ultra-fine fly ash fraction demonstrates superior performance in pore structure refinement due to its high specific surface area and excellent particle size compatibility with hydration products, resulting in a more compact microstructure with improved durability characteristics. These findings underscore the importance of particle size control in engineering the pore architecture of fly ash-modified cementitious materials for enhanced performance.

#### 3.3.3. Effect of Fly Ash Particle Size on Pore Size Distribution of Mortar

**Figure 14:**
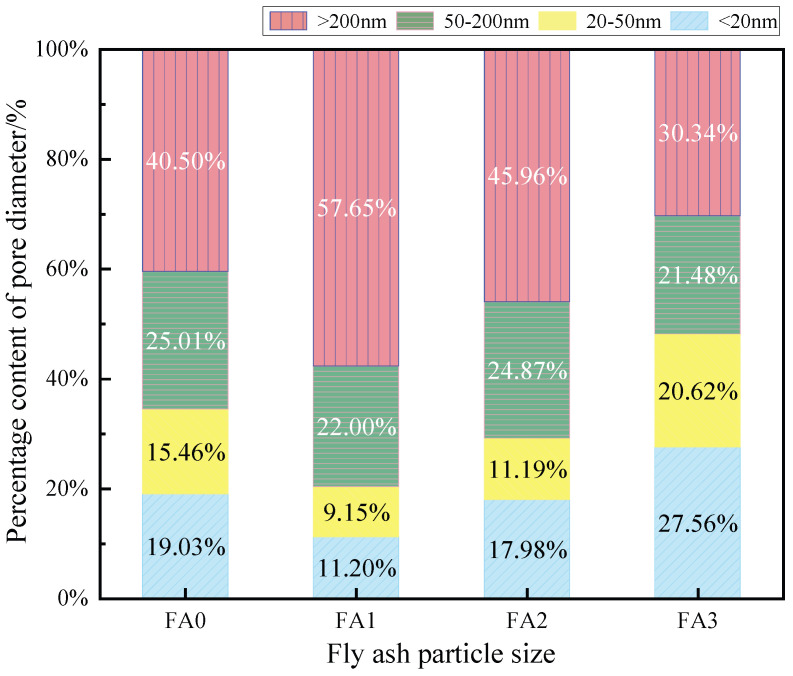
Percentage of each pore diameter of cement mortar mixed with fly ash of different particle sizes.

Figure 14 illustrates the pore size distribution of cement mortars incorporating fly ash with different particle sizes (FA0, FA1, FA2, and FA3). The results show that the content of harmless pores (less than 20 nm) in FA0 mortar is 19.03%, while FA1 mortar exhibits a lower content of 11.20%, representing a 7.83% reduction compared to FA0. In contrast, FA2 mortar shows a slight decrease of 1.05%, whereas FA3 mortar demonstrates a significant increase of 8.53%. For the less harmful pore range (20–50 nm), FA0 mortar contains 15.46%, while FA1, FA2, and FA3 mortars show changes of −6.31%, −4.27%, and +5.16%, respectively.

The harmful pore (50–200 nm) content in FA0 mortar is 25.01%, with FA1, FA2, and FA3 mortars exhibiting reductions of 3.01%, 0.14%, and 3.53%, respectively. Notably, the most significant differences are observed in the multi-harmful pore range (greater than 200 nm), where FA0 mortar contains 40.50%, while FA1 mortar shows a substantial increase of 17.46%. FA2 and FA3 mortars demonstrate changes of +5.46% and −15.16%, respectively.

Overall, finer fly ash particles effectively optimize the pore structure distribution by increasing the proportion of harmless and less harmful pores while reducing harmful and multi-harmful pores. Medium-sized particles exhibit minimal modifications compared to the control, with slight reductions in beneficial pores and minor increases in detrimental pores. Conversely, coarser particles significantly decrease the content of advantageous pores while substantially elevating the proportion of multi-harmful pores. These findings clearly demonstrate that fly ash particle size plays a crucial role in determining the pore structure characteristics of cement mortar, thereby significantly influencing its overall performance. The results highlight the importance of particle size optimization for achieving desired microstructural properties in fly ash-modified cementitious materials.

### 3.4. Effect of Fly Ash Particle Size on the Phase of Mortar

#### 3.4.1. XRD Analysis

**Figure 15:**
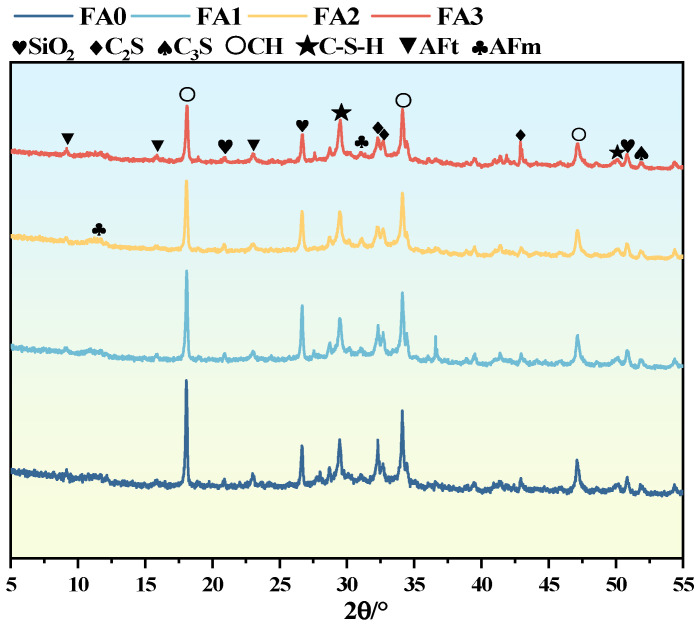
XRD pattern of mortar by fly ash with different particle sizes at 28 days.

Figure 15 presents the XRD analysis of mortar made with fly ash of different particle sizes at age of 28 days. Despite the variation in fly ash particle size, the main hydration products in the samples are consistently calcium hydroxide (CH), Aft, C-S-H gel, and AFm. This indicates that the particle size of fly ash does not alter the types of hydration products, but it does lead to differences in their quantities. Mortar made with fly ash of different particle sizes still has unhydrated C_2_S and C_3_S after 28 days curing. As fly ash particle size decreases, the XRD diffraction peaks of AFt, C-S-H gel, and AFm gradually strengthen, while the peaks of CH and C_2_S, C_3_S in cement clinker weaken. This shows that smaller fly ash particle size promotes mortar hydration. As fly ash particle size decreases, its specific surface area increases. This promotes a secondary hydration reaction between active components in fly ash and hydration products like calcium hydroxide in mortar. More C-S-H gel is formed and fills mortar pores. Also, smaller fly ash particles better fill these pores, improving the pore structure, reducing porosity, and enhancing mortar strength and density. Thus, compressive strength tends to rise with smaller fly ash particle size (Figure 15). The reduction in CH content may be due to the increased surface energy and pozzolanic activity of finer fly ash particles, which promotes the pozzolanic reaction and consumes CH to form C-S-H gel. The presence of SiO_2_ was also observed in the hydration products of fly ash with different particle sizes, which was analyzed as the main mineral phase of fly ash according to XRD.

#### 3.4.2. TG/DSC Analysis

**Figure 16:**
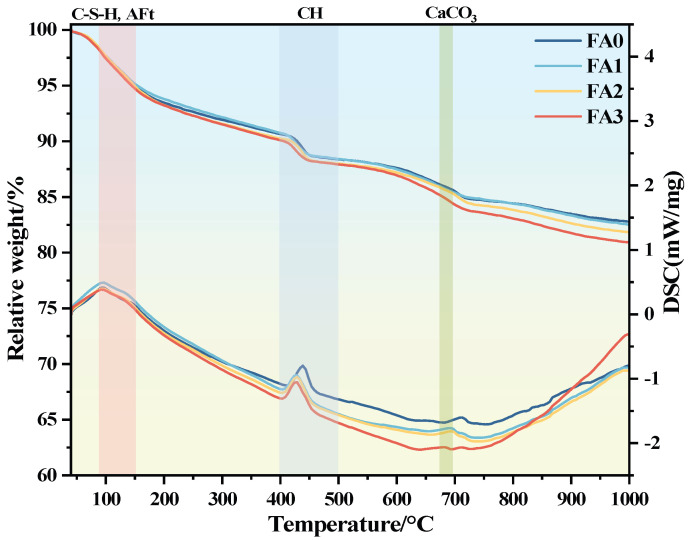
TG/DSC pattern of mortar by fly ash with different particle sizes at 28 days.

Figure 16 presents TG and DSC curves for mortar made with fly ash of different particle sizes at a curing age of 28 days. The mass of all the samples showed a decreasing trend as the temperature increased, and a clear weight loss interval was observed from 90 °C to 150 °C, and there was a clear heat absorption peak in the DSC curve. This is mainly due to the evaporation of free water in hydration products and the thermal decomposition of C-S-H and AFt. While C-S-H gel can indicate the degree of mortar hydration, its weight loss zone overlaps with that of AFt, making it difficult to distinguish. A second weight loss peak was observed between 400 °C and 500 °C, corresponding to an endothermic peak on the DSC curve. This is mainly due to the thermal decomposition of CH, a hydration product. Normally, CH content correlates with the amount of hydration products and can indirectly indicate the degree of mortar hydration. However, the gel material was doped with fly ash, and Ca(OH)_2_ to generate a C-S-H gel, as a result, CH content no longer has a direct proportional relationship with the degree of hydration. However, as the particle size of fly ash decreases, the total weight loss of the sample gradually increases. This indicates that the reduction in particle size does not change the types of hydration products but causes a difference in their content and promotes mortar hydration. The weight loss peak and endothermic peak at 680 °C are due to the decomposition of CaCO_3_ into CaO and CO_2_. The smaller the fly ash particle size, the greater the specific surface area, which leads to a more complete pozzolanic reaction and further promotes mortar hydration.

The smaller the particle size of fly ash, the larger its specific surface area, which increases the contact area with water and thereby leads to a greater amount of hydration products formed. This further explains that in the hydration exothermic test, as the particle size of fly ash decreases, the heat release of the mortar increases. Specifically, the smaller fly ash particles (FA3) promote the hydration process of concrete, accelerate the reaction kinetics, and facilitate the formation of more gel-like substances such as C-S-H. In contrast, the coarser fly ash particles (FA1) reduce surface reactivity, retard the reaction kinetics, and result in a decrease in the content of hydration products. Consequently, the total heat release is reduced.

### 3.5. Analysis of Microstructure of Cement Mortar

**Figure 17:**
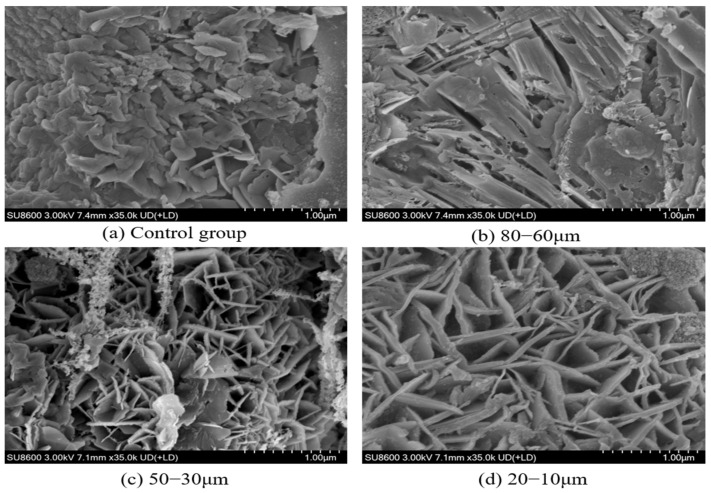
The 28 days micro-morphology of cement mortar.

Figure 17 presents the scanning electron microscopy (SEM) analysis of cement mortars incorporating different fly ash particle sizes (unsieved control, 80–60 μm, 50–30 μm, and 20–10 μm) after 28 days of curing. The upper images show the internal microstructure of cement-fly ash composite mortars containing 10% fly ash replacement.

As observed in Figure 17a, the control mortar (without fly ash) exhibits abundant stacked hexagonal plate-like calcium hydroxide (CH) crystals with limited needle-like calcium silicate hydrate (C-S-H) gel formation on their surfaces after 28 days of hydration. Figure 17b reveals the microstructure of mortar containing 80–60 μm fly ash, demonstrating increased formation of needle-shaped C-S-H gel. This enhancement results from the progressive consumption of CH crystals by reactive SiO_2_ from fly ash particles, leading to additional C-S-H gel formation within pore spaces.

Figure 17c,d display the microstructures of mortars incorporating finer fly ash fractions (50–30 μm and 20–10 μm, respectively). Compared with Figure 17b, these specimens show significantly denser C-S-H gel networks with more pronounced needle-like morphology. The refined microstructural development confirms that finer fly ash particles effectively fill internal voids while promoting extensive pozzolanic reactions, thereby substantially improving the overall compactness of the cement matrix. These microscopic observations correlate well with the enhanced mechanical performance observed in finer fly ash-modified mortars, demonstrating the critical role of particle size in microstructural optimization of cementitious composites.

### 3.6. Discussion

**Figure 18:**
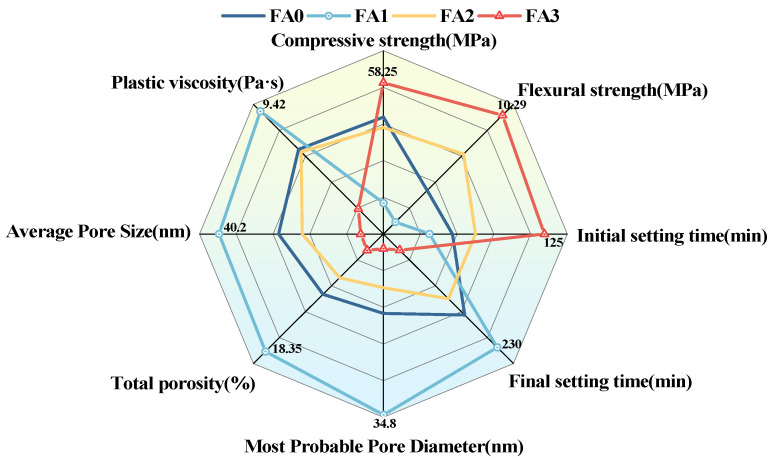
Analysis diagram of the influence of particle size on different properties of fly ash cement.

Figure 18 presents the performance of mortars with different fly ash particle size groups (FA0, FA1, FA2, FA3) in multiple indicators, including compressive strength, flexural strength, initial setting time, final setting time, most probable pore diameter, total porosity, and average pore diameter. When cement mortar was prepared using fly ash with the smallest particle size (FA3), the compressive and flexural strengths of the specimens were improved, the initial setting time was prolonged, the final setting time was shortened, and the most probable pore diameter, total porosity, and average pore diameter were reduced, thereby enhancing the compactness of the cement mortar. However, the performance of cement mortar prepared with fly ash of medium particle sizes (FA1, FA2) was lower than that of the control group (FA0). It is speculated that FA2 and FA3 may have accidentally accumulated a higher proportion of hydration-inert phases, or the content of unburned carbon in them was relatively higher. These inert components do not participate in the hydration reaction, leading to the decrease in performance.

## 4. Conclusions

In this study, fly ash with different particle size distributions was used to replace cement to prepare mortar, and the effect of particle size distribution on the properties of cement mortar was studied. The main conclusions are as follows:

(1) Fly ash fractions within different particle size ranges have the same composition, mainly consisting of SiO_2_, Al_2_O_3_, CaO, CaCO_3_, and CaSO_4_. All fly ash samples with varying particle sizes exhibit both irregular and spherical morphologies; however, the sieved fly ash shows more regular shapes and a more uniform distribution. Additionally, as the particle size of fly ash decreases, the light transmittance of Si–O bonds and Al–O bonds decreases.

(2) As the particle size of fly ash decreases, it prolongs the initial setting time of mortar, shortens its final setting time, and increases the mortar’s compressive strength and flexural strength. Fly ash with different particle sizes has little impact on the total heat release during the early stage of cement hydration. As the hydration time increases, the total amount of hydration heat release rises. The smaller the particle size of fly ash, the larger its specific surface area. This enables the pozzolanic effect to occur, thereby promoting the hydration reaction of cement mortar.

(3) As the particle size of fly ash decreases, the yield stress and plastic viscosity of the mortar exhibit a trend of first increasing and then decreasing, while the torque and shear stress show a tendency of first decreasing and then increasing. Furthermore, the total porosity, average pore diameter, and most probable pore diameter of the mortar decrease as the particle size of fly ash decreases. This optimizes the pore structure, reduces harmful pores, and increases harmless pores, thereby improving the compactness of the mortar.

(4) After 28 days of curing, the types of hydration products in mortars incorporating fly ash with different particle sizes remain unchanged. The smaller the particle size of fly ash, the larger its specific surface area, which leads to a more complete pozzolanic reaction and thereby promotes the hydration of the mortar. SEM analysis reveals that the incorporation of fly ash with a particle size of 10–20 μm can promote the formation of C-S-H gel in cement mortar, fill pores, and thereby enhance the compactness of the cement mortar.

## Figures and Tables

**Table 1:** Chemical composition of cement (mass fraction)/%.

Chemical Composition	CaO	Fe_2_O_3_	Al_2_O_3_	MgO	SiO_2_	SO_3_	Loss on Ignition
Content	61.17	4.52	7.22	4.63	22.35	2.34	5.31

**Table 2:** Basic properties of cement.

Setting Time (min)		Compressive Strength (MPa)		Flexural Strength (MPa)	
Initial Setting Time	Final Setting Time	3 Days	28 Days	3 Days	28 Days
125	175	25.4	55.3	4.6	7.5

**Table 3:** Chemical composition analysis of fly ash/%.

Chemical Composition	CaO	SiO_2_	Fe_2_O_3_	Al_2_O_3_	MgO	SO_3_	Miscellaneous
Content	4.73	52.68	7.58	27.77	1.71	1.23	1.84

**Table 4:** Physical properties of fly ash.

Density (g/cm^3^)	Specific Surface Area (m^2^/kg)	Water Demand (%)	Loss on Ignition (%)	Active Index (%)
2.76	438	90	3.0	82.6

**Table 5:** The test cement mortar mix ratio Kg/m^3^.

Numbering	Fly Ash Particle Size	W/C	Cement	Water	Fly Ash	River Sand
FA0	Control group	0.4	850	380	94	1416
FA1	60–80 μm					
FA2	30–50 μm					
FA3	10–20 μm					

Note: FA0 is a cement mortar prepared using untreated fly ash.

**Table 6:** Comparison table of mesh number and corresponding pore size in this test.

Mesh Number	Aperture/μm
180	80
250	60
300	50
500	30
800	20
1400	10

**Table 7:** Rheological change trend diagram of fly ash with different particle sizes.

Fly Ash Particle Size	Yield Stress/Pa	Plastic Viscosity/Pa·s
FA0	336.31	8.98
FA1	313.91	8.29
FA2	316.16	8.95
FA3	385.26	9.42

## Data Availability

The original contributions presented in this study are included in the article. Further inquiries can be directed to the corresponding author.
